# Species-differences in the in vitro biotransformation of trifluoroethene (HFO-1123)

**DOI:** 10.1007/s00204-023-03603-3

**Published:** 2023-10-04

**Authors:** R. Dekant, R. Bertermann, J. Serban, S. Sharma, M. Shinohara, Y. Morizawa, H. Okamoto, W. Brock, W. Dekant, A. Mally

**Affiliations:** 1 Department of Toxicology, University of Würzburg, Versbacher Strasse 9, 97078 Würzburg, Germany; 2 Department of Inorganic Chemistry, University of Würzburg, Am Hubland, 97074 Würzburg, Germany; 3 Chemicals Company, AGC Inc, CSR Office, 1-5-1, Marunouchi, Chiyoda-ku, Tokyo, 100-8405 Japan; 4 Brook Scientific Consulting LLC, Hilton Head Island, SC USA

**Keywords:** Trifluoroethene, HFO-1123, Mercapturic acid pathway, In vitro biotransformation, β-Lyase, ^19^F-NMR, LC–MS/MS

## Abstract

1,1,2-Trifluoroethene (HFO-1123) is anticipated for use as a refrigerant with low global warming potential. Inhalation studies on HFO-1123 in rats indicated a low potential for toxicity (NOAELs ≥ 20,000 ppm). In contrast, single inhalation exposure of Goettingen^®^ minipigs (≥ 500 ppm) and New Zealand white rabbits (≥ 1250 ppm) resulted in severe toxicity. It has been suggested that these pronounced species-differences in toxicity may be attributable to species-differences in biotransformation of HFO-1123 via the mercapturic acid pathway. Therefore, the overall objective of this study was to evaluate species-differences in glutathione (GSH) dependent in vitro metabolism of HFO-1123 in susceptible versus less susceptible species and humans as a basis for human risk assessment. Biotransformation of HFO-1123 to *S*-(1,1,2-trifluoroethyl)-*L*-glutathione (1123-GSH) and subsequent cysteine S-conjugate β-lyase-mediated cleavage of the corresponding cysteine conjugate (1123-CYS) was monitored in hepatic and renal subcellular fractions of mice, rats, minipigs, rabbits, and humans. While 1123-GSH formation occurred at higher rates in rat and rabbit liver S9 compared to minipig and human S9, increased β-lyase cleavage of 1123-CYS was observed in minipig kidney cytosol as compared to cytosolic fractions of other species. Increased β-lyase activity in minipig cytosol was accompanied by time-dependent formation of monofluoroacetic acid (MFA), a highly toxic compound that interferes with cellular energy production via inhibition of aconitase. Consistent with the significantly lower β-lyase activity in human cytosols, the intensity of the MFA signal in human cytosols was only a fraction of the signal obtained in minipig subcellular fractions. Even though the inconsistencies between GSH and β-lyase-dependent metabolism do not allow to draw a firm conclusion on the overall contribution of the mercapturic acid pathway to HFO-1123 biotransformation and toxicity in vivo, the β-lyase data suggest that humans may be less susceptible to HFO-1123 toxicity compared to minipigs.

**Supplementary Information:**

The online version contains supplementary material available at 10.1007/s00204-023-03603-3.

## Introduction

As hydrofluorocarbons (HFCs) currently used as refrigerants in domestic and mobile air-conditioning and refrigeration are currently being phased out by the Kigali Amendment to the Montreal Protocol (Heath 2017), there is a continuing need for introducing novel, climate friendly refrigerants. 1,1,2-Trifluoroethene (HFO-1123, CAS# 359-11-5), which exhibits zero ozone depleting potential and a low global warming potential of 0.3 (1/3 of the greenhouse effect of CO_2_) while retaining identical cooling efficiency as currently used HFCs, has been identified as a promising candidate compound for air-conditioning units (AGC Inc. 2022).

Inhalation studies on HFO-1123 in rodent and non-rodent species revealed marked species-differences in toxicity. Nose-only single-dose inhalation exposure of Sprague–Dawley^®^ (SD) rats to HFO-1123 at concentrations up to 200,000 ppm did not cause clinical signs or histopathological evidence of toxicity (Wako 2014b). A repeated dose-toxicity inhalation study in rats with HFO-1123 for 14 days (6 h/day, 5 days/week) at concentrations up to 20,000 ppm also suggested a low toxicity potential of HFO-1123. From this study, a no-observable-adverse-effect-level (NOAEL) ≥ 20,000 ppm was derived (Wako 2014a). In contrast to rats, single-dose inhalation exposure of Goettingen^®^ minipigs and New Zealand white rabbits resulted in mortality at concentrations ≥ 500 ppm and ≥ 1250 ppm, respectively (Wako 2016, 2017). In minipigs exposed to HFO-1123 at concentrations > 65 ppm, emesis, convulsion, locomotor activity disorders, significantly lower neutrophil and higher values of troponin in serum were observed (Wako 2018b). Minipigs that died during exposure presented hemorrhage and focal inflammatory cell infiltration in the epicardium and interstitium, thickening of the intramural artery, and increased pericardial fluid in the pericardial cavity. Similarly, focal white changes of the heart, pulmonary edema, and hydrothorax, and dark reddish patches in the lungs were observed in rabbits.

**Fig. 1:**
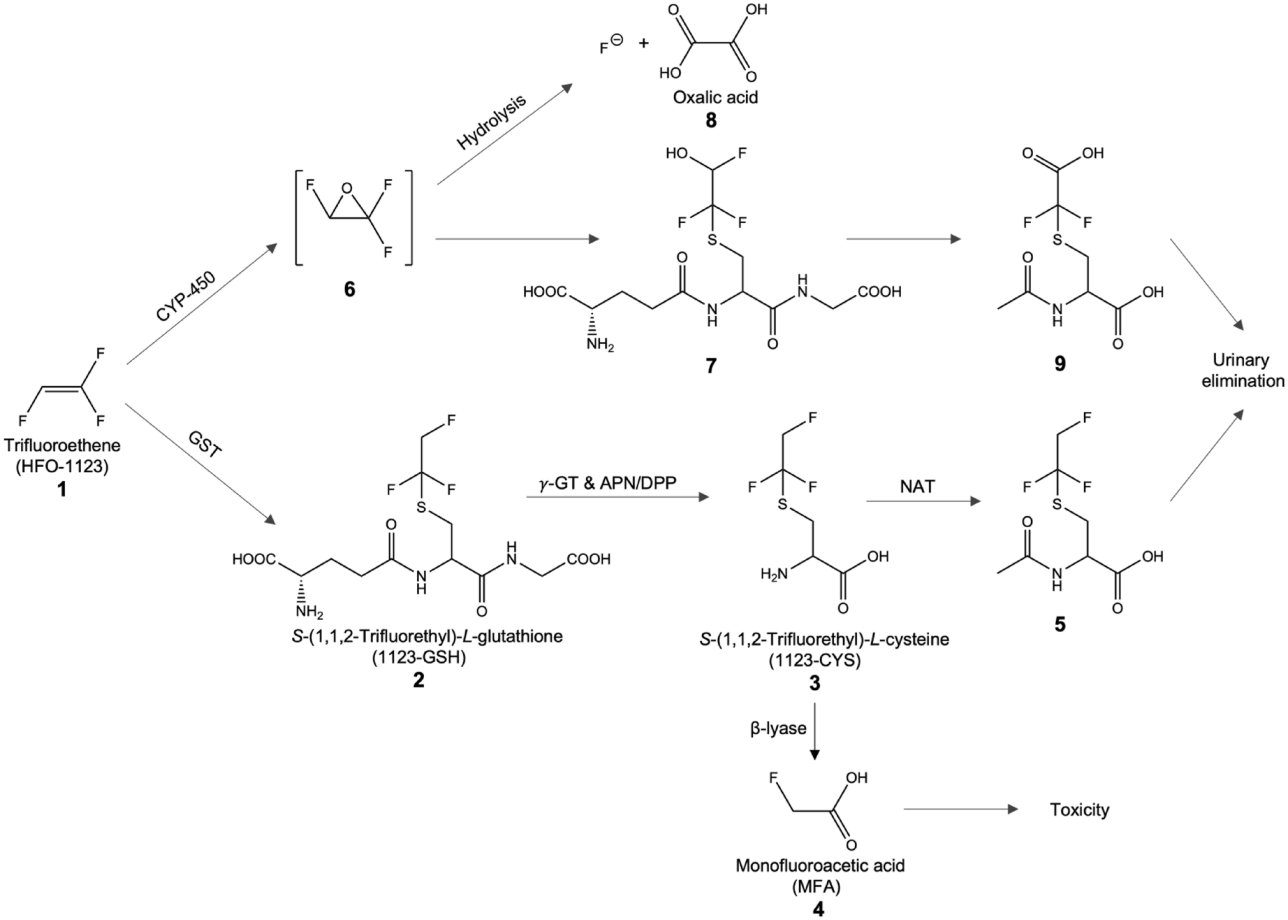
Proposed biotransformation of HFO-1123 **1** to account for its toxicity in sensitive species. Cytochrome P450 (CYP-450)-mediated biotransformation may result in hydrolysis of the 1,2,2-trifluorooxirane **6** into F^−^ and oxalic acid **8** or subsequent conjugation with GSH **7** and urinary excretion of a mercapturic acid **9**. The mercapturic acid pathway (lower pathway) is expected to be responsible for HFO-1123 toxicity. HFO-1123 may be conjugated with GSH, likely mediated by glutathione *S*-transferases (GST), to form *S*-(1,1,2-trifluorethyl)-*L*-glutathione (1123-GSH) **2**. This conjugate may be cleaved by γ-glutamyltransferases (γ-GT) and aminopeptidase (APN)/dipeptidase (DPP) to form the corresponding cysteine *S*-conjugate **3** (*S*-(1,1,2-trifluoroethyl)-*L*-cysteine; 1123-CYS). 1123-CYS can either be eliminated by *N*-acetyltransferases (NAT) or bioactivated by β-lyases. The latter reaction may give rise to monofluoroacetic acid **4** (MFA) as the ultimate toxic agent expected to be responsible for HFO-1123 toxicity

Considering the marked species-differences in inhalation toxicity of HFO-1123, it is hypothesized that the higher sensitivity of minipigs and rabbits as compared to rats may be linked to species-differences in biotransformation of HFO-1123. This biotransformation differences may result in the formation of toxic metabolites in susceptible species. Based on the metabolism of structurally related haloalkenes, it was anticipated that both oxidative metabolism mediated by cytochrome P450 enzymes (CYP-450) as well as direct coupling with glutathione (GSH) may contribute to biotransformation of HFO-1123 (Fig. 1) (Schuster et al. 2009; Schmidt et al. 2013; Lash et al. 2014). CYP-450-mediated oxidation of HFO-1123 **1** may generate an intermediate epoxide (1,2,2-trifluorooxirane) **6**, which may undergo hydrolysis to fluoride and oxalic acid **8** or may further react with GSH in an enzymatic reaction mediated by glutathione *S*-transferases (GSTs) to form a GSH conjugate **7**. Subsequent processing of the GSH conjugate to its corresponding cysteine conjugate may lead to renal excretion in the form of the corresponding mercapturic acid **9**. While these CYP-450-dependent metabolites of HFO-1123 are unlikely to play a significant role in HFO-1123 toxicity, biotransformation of HFO-1123 via the mercapturic acid pathway may give rise to monofluoroacetic acid (MFA)** 4**, a highly toxic metabolite. This bioactivation pathway is initiated by direct conjugation of HFO-1123 with GSH, likely mediated by GSTs, to form *S*-(1,1,2-trifluorethyl)-*L*-glutathione (1123-GSH) **2**, which may be enzymatically cleaved to the corresponding cysteine *S*-conjugate, *S*-(1,1,2-trifluorethyl)-*L*-cysteine **3** (1123-CYS), via γ-glutamyltransferases (γ-GT) and aminopeptidases N (APN). Subsequent *N*-acetylation of 1123-CYS may yield a mercapturic acid (*N*-acetyl-cysteine *S*-conjugate) derivative **5**, which may be excreted via urine. However, 1123-CYS may also be a substrate for cysteine *S*-conjugate β-lyases (β-lyases) mediated cleavage, which may result in the formation of MFA **4**. β-Lyases represent a heterogeneous group of enzymes that catalyze β-eliminations of cysteine *S*-conjugates. Differences in expression of different β-lyases between tissue and species exist and may influence activity and substrate specificity (Cooper and Pinto 2006).

Due to inhibition of the enzyme aconitase in the tricarboxylic acid (TCA) cycle, MFA is highly toxic (Goncharov et al. 2006; Johnson et al. 2020). Inhibition of the TCA cycle by MFA can lead to a multitude of biochemical changes, including lactate acidosis, accumulation of citrate, hypocalcemia, glutamine depletion, ketosis, and adenosine triphosphate (ATP) depletion, which may result in cardiac arrest, ventricular fibrillation, convulsion, and central nervous system depression, ultimately leading to death (Proudfoot et al. 2006; Eason et al. 2011). Thus, the clinical and histopathological signs of toxicity observed in the deceased minipigs and rabbits exposed to HFO-1123, characterized by cardiac and CNS toxicity, are consistent with symptoms of MFA poisoning and point towards a role of MFA in HFO-1123 toxicity in susceptible species.

The objective of the present study was to experimentally support the hypothesis that species-differences in the extent of GSH-dependent biotransformation and subsequent β-lyase-mediated formation of MFA may account for the species-differences in HFO-1123 toxicity by investigating HFO-1123 biotransformation in subcellular fractions of susceptible (minipig, rabbit) versus less susceptible species (rats, mice) and humans to improve the basis for human risk assessment.

## Materials and methods

### Chemicals and subcellular fractions

*S*-(1,2,2-Trichlorovinyl)-*L*-cysteine (TCVC) and monofluoroacetic acid (MFA) were purchased from Toronto Research Chemicals (North York, Canada). HFO-1123 (purity ≥ 99%) and crude 1123-CYS were supplied by AGC Inc. (Tokyo, Japan). 1123-CYS was further purified by HPLC. The purity of 1123-CYS was characterized by HPLC–DAD, LC/MS–MS, and ^19^F-NMR and determined to be > 97%. Unless otherwise indicated, chemicals were purchased from Merck Millipore (Darmstadt, Germany), ThermoFisher Scientific (Dreieich, Germany), or Roth (Karlsruhe, Germany) in the highest purity available.

Liver and kidney S9 fractions from SD rats (liver: male, pool of 97 donors; kidney: male, pool of 150 donors), humans (liver: mixed gender, pool of 50 donors; kidney: mixed gender, pool of 8 donors), and hepatic S9 from CD1 mice (male, pool of 1745 donors) were obtained from XenoTech^®^ (Lenexa, USA). Hepatic and renal cytosolic fractions from humans (kidney: pool of 4 donors, mixed gender; liver: pool of 10 donors, male), SD rats (kidney: 150 donors, male; liver: 454 donors, male), and CD1 mice (kidney: pool of 250 donors; liver: pool of 1200 donors, male) were also obtained from XenoTech^®^ (Lenexa, USA). Renal and hepatic cytosolic and S9 fractions were prepared from a Goettingen^®^ minipig (male) and a NZW rabbit (male) based on the standard protocols (Hubbard et al. 1985; Dekant et al. 1998). Organs from a Goettingen® minipig (sex: male; bodyweight: 42.4 kg; Date of birth: 24.07.2015; Date of sacrifice: 13.05.2020; Identification Nr. 23428) were obtained from the *Department of Animal Sciences* of the University of Goettingen (Goettingen, Germany). The liver and kidneys of a male New Zealand White rabbit were kindly provided by the *Department for Functional Materials in Medicine and Dentistry* of the University of Wuerzburg.

### Chemical synthesis

#### Synthesis of S-(1,1,2-trifluoroethyl)-l-glutathione (1123-GSH)

*S*-(1,1,2-trifluoroethyl)-*L*-glutathione (1123-GSH) was synthesized according to McKinney et al*.* (McKinney et al. 1957) with minor modifications. Reactions were carried out at − 80 °C (cooled by a mixture of methanol/CO_2_). Sodium (3 mmol) was dissolved in liquefied ammonia (50 mL). To this solution, *L*-glutathione (3 mmol) was added and dissolved. Finally, an excess of gaseous HFO-1123 was condensed into the flask and the obtained solution was stirred for 5 h at − 80 °C. The reaction was stopped by removing the reaction flask from the cooling source. The reaction mixture was then stored at 25 °C overnight in a fume hood to evaporate remaining HFO-1123 and NH_3_. The obtained crystal residue was dissolved in water and acidified to pH 3 with 2 N hydrochloric acid. The synthesis product was purified by HPLC and characterized by Enhanced Product Ion (EPI) spectrum. LC–MS/MS*: m/z*: 388 ([M -H^+^]^−^), 272 ([M -H^+^ -HSC_2_F_3_H_2_]^−^), 254 ([M -H^+^ -HSC_2_F_3_H_2_ -H_2_O]^−^), 210 ([M -H^+^ -HSC_2_F_3_H_2_ -H_2_O -CO_2_]^−^), 179 ([M -H^+^ -HSC_2_F_3_H_2_ -H_2_O -glycine]^−^), 143 ([M -HSC_2_F_3_H_2_ -HCO(C_3_H_5_)NH_2_COO]^−^), 128 ([M -HSC_2_F_3_H_2_ -HCO(C_3_H_5_)NH_2_COO -NH_2_]^−^), 115 ([M -H^+^ -C_10_H_15_N_3_O_6_]^−^), 95 ([M -H^+^ -C_10_H_15_N_3_O_6_ -HF]^−^). The structure was confirmed by ^1^H-; ^19^F-; ^19^F{^1^H}-; ^19^F-,^19^F COSY-; ^19^F,^1^H HMQC-; ^13^C{^1^H}-; ^13^C DEPT135-; ^13^C DEPT90-; ^13^C{^19^F}-; ^13^C-,^19^F HMQC-; ^1^H,^1^H COSY-; ^13^C,^1^H HSQC-; ^13^C,^1^H HMBC-; ^1^H- with water suppression and ^1^H DOSY-NMR. NMR analysis revealed *S*-*trans*-(1,2-difluoroethenyl)-*L*-glutathione as an impurity. ^1^H-NMR of 1123-GSH/*S*-*trans*-(1,2-difluoroethenyl)-*L*-glutathione (500 MHz, D_2_O): δ = 2.10 ppm (m, 4H), δ = 2.47 ppm (m, 4H), δ = 3.04 ppm (dd, 1H), δ = 3.15 ppm (dd, 1H), δ = 3.29 ppm (dd, 1H), δ = 3.38 ppm (dd, 1H), δ = 3.74 (t, 1H), δ = 3.75 ppm (t, 1H), δ = 3.89 ppm (s, 2H), δ = 3.90 ppm (s, 2H), δ = 4.57 ppm (dd, 2H), δ = 4.71 ppm (m, H_2_O), δ = 4.68 ppm (m, 2H), δ = 7.57 ppm (d, 1H). ^13^C-NMR of 1123-GSH/*S*-*trans*-(1,2-difluoroethenyl)-*L*-glutathione (125 MHz, D_2_O): δ = 25.96, 28.78, 31.18, 32.88, 41.56, 53.29, 53.77, 81.90, 126.51, 147.10, 171.79, 171.95, 173.56, 174.82 ppm. ^19^F-NMR of 1123-GSH/*S*-*trans*-(1,2-difluoroethenyl)-*l*-glutathione (470 MHz, D_2_O): δ = − 227.85 ppm (m, 1F), − 157.20 ppm (dd, 1F), − 140.33 ppm (d, 1F), − 85.02 ppm (m, 2F). Corresponding NMR spectra are available in the supplementary material, Fig. S1 and S2. Quantitative ^19^F-NMR spectra revealed a ratio of 1:0.875 for 1123-GSH and *S*-*trans*-(1,2-difluoroethenyl)-*l*-glutathione. Further separation of both compounds by preparative HPLC was not possible. However, the ratio of 1123-GSH to *S*-*trans*-(1,2-difluoroethenyl)-*l*-glutathione enabled determination of the amount of 1123-GSH in the mixture obtained by HPLC.

#### Synthesis of S-(1,1,2-trifluoroethyl)-L-cysteine (1123-CYS)

*S*-(1,1,2-trifluoroethyl)-*L*-cysteine (1123-CYS) was synthesized according to Sachdev et al. (Sachdev et al. 1990) with the following modifications. *N*-Acetyl-*L*-cysteine (3.1 mmoles) was dissolved in H_2_O (16 ml), MeOH (18 ml), and sodium methoxide (in MeOH, 5 mol/L, 3.1 mmoles) in autoclave. Gaseous HFO-1123 (ca. 4 g) was introduced and the obtained solution was stirred for 18 h at 45–49 °C and an internal pressure of 0.40 MPa. Subsequently, the volatiles were evaporated under reduced pressure at 40–50 °C. The obtained residue was cooled in an ice bath and acidified with diluted hydrochloric acid followed by the extraction with ethyl acetate. After drying with MgSO_4_, the solvent was evaporated to obtain the crude product (2.37 g) as a colorless viscous liquid. 1.15 g of the crude product was treated with 6N hydrochloric acid (5 ml) at 80 °C for 8 h and dried under reduced pressure. Conformation that racemization did not occur was performed by comparison of chiral HPLC chromatograms (performed with a Daicel CROWNPACK CR-I ( +)) and mass spectra of the corresponding *D*-form product. The obtained dried synthesis product was further purified by HPLC and characterized by EPI. LC–MS/MS: *m/z*: 202 ([M -H^+^]^−^), 115 ([M -H^+^ -CH_2_CNH_2_COOH]^−^], 95 ([M -H^+^ -CH_2_CNH_2_COOH -HF]^−^]. The structure was confirmed by ^19^F-NMR (supplementary material, Fig. S3). ^19^F-NMR (470 MHz, D_2_O): δ = -228.28 ppm (m, 1F), − 84.61 ppm (m, 2F).

### Instrumental analyses

#### ^19^F-NMR analysis

^19^F-NMR spectra were recorded in D_2_O at 25 °C on a Bruker 500 MHz NMR spectrometer (Bruker Corporation, Billerica, USA) equipped with a 5 mm BB/H&F Prodigy Cryoprobe operating at 470.59 MHz. ^19^F chemical shifts were referenced to external CFCl_3_. The acquisition time was set to 0.799 s and 5120 scans were recorded. Spectral processing was performed with 0.05 Hz line broadening. ^19^F-NMR spectra were acquired without proton decoupling and a spectral width of 212.5 ppm (− 33.4 to − 245.9 ppm). Before analysis, accurate baseline correction and spectra phasing were performed. NMR spectra were analyzed with TopSpin 4.1.1 software (Bruker Corporation, Billerica, USA) for MAC OS 11.5.2.

#### LC–MS/MS analyses

LC–MS/MS analyses were performed on an Agilent 1100 series LC (Agilent, Waldbronn, Germany) coupled to an API 2000/Q-Trap mass spectrometer (Applied Biosystems/MDS Sciex, Darmstadt, Germany). Samples were injected into the LC–MS/MS system through an Agilent 1100 series autosampler. Separations were carried out on a ReproSil-Pur C18 AQ column (2 mm × 150 mm, 5 µm; Dr. Maisch; Ammerbuch, Germany). Gradient elution was carried out with water + 0.1% formic acid (FA) (solvent A) and acetonitrile + 0.1% FA (solvent B). Initially, solvent A was held isocratic for 5 min at 100%, followed by a linear gradient to 95% B in 5 min. These conditions were held for further 5 min. Within 1 min, the gradient decreased linearly to 0% B and remained at initial conditions until the end of analysis (30 min). A flow rate of 0.2 mL/min was used. For each run, 10 µL of the respective sample were injected by the autosampler. The API 2000/Q-Trap mass spectrometer was operated with a Turbo Ion Spray source in positive-ion mode with a voltage of + 4000 V. Spectral data were recorded with N_2_ as the heater gas at 450 °C and as the collision gas (CAD = 2) in multiple reaction monitoring mode (MRM). The following MRM parameters for quantitation and identification of HFO-1123-derived metabolites were used: m/z 1123-GSH (Q1 → Q3): 390.1 → 221.0, 390.1 → 175.0; m/z 1123-CYS (Q1 → Q3): 203.8 → 118.1, 203.8 → 164.0. Enhanced product ion spectra were recorded over the range of *m/z* 80–400 in negative- or positive-ion mode. The collision gas was N_2_ at CAD = 2, and the collision energy was 30 V. Method validation parameters, such as limit of detection (LOD) and quantification (LOQ), were determined according to European Commission Regulation 657/2002/EC (European Commission 2002). The LOD and LOQ of 1123-GSH and the LOD of 1123-CYS were determined by serial dilution of S9 subcellular fractions spiked with 1123-GSH and 1123-CYS. LODs and LOQs were obtained using a signal-to-noise (S/N) ratio of 3:1 and 7:1, respectively. 1123-GSH: LOD: 0.013 µM (5 ng/mL), LOQ: 0.025 µM (10 ng/mL); 1123-CYS: LOD: 0.005 µM (1 ng/mL). Calibration for 1123-GSH was linear in the range from 10 ng/mL to 800 ng/mL (R^2^ values ≥ 0.99).

### In vitro biotransformation

#### Sample preparation for GSH-dependent biotransformation of HFO-1123 by LC–MS/MS and ^19^F-NMR

To study time-dependent formation of 1123-GSH, HFO-1123 (LC–MS/MS: 0.4 mmol; ^19^F-NMR: 0.89 mmol) was incubated with S9 subcellular fractions (3 mg protein/mL) of minipigs, rabbits, rats, mice, and humans in sealed 20 mL GC vials (Supelco^®^ Inc, Bellefonte, USA). Incubations (total volume of 1.1 mL) were carried out in the presence of GSH (10 mM) with and without an NADPH regenerating system (10 mM NADP, 10 mM glucose-6-phosphate and 0.5 units/mL glucose-6-phosphate dehydrogenase) in potassium phosphate buffer (100 mM, pH 7.4, containing 1 mM EDTA). Additional incubations containing GSH and hepatic S9 were performed in the presence of the specific γ-GT inhibitor acivicin (0.5 mM) to evaluate potential γ-GT-mediated cleavage of 1123-GSH or in the presence of AOAA (5 mM), a specific β-lyase inhibitor, to evaluate potential β-lyase-mediated cleavage of 1123-CYS which may be formed from 1123 to GSH.

To determine in vitro kinetics of the formation of 1123-GSH, hepatic S9 subcellular fraction (3 mg protein/mL) of minipigs, rabbits, rats, mice, and humans were incubated with 0.18 mmoles (4 mL), 0.27 mmoles (6 mL), 0.36 mmoles (8 mL), 0.45 mmoles (10 mL), 0.53 mmoles (12 mL), and 0.89 mmoles (20 mL) gaseous HFO-1123 injected via a gas-tight Hamilton syringe into the gas phase of sealed 20 ml GC vials. Incubations (total volume of 1.1 mL) were carried out in the presence of GSH (10 mM) in potassium phosphate buffer (100 mM, pH 7.4, containing 1 mM EDTA).

Incubations were carried out in a shaking water bath at 37 °C for up to 12 h. For LC–MS/MS analyses, aliquots (200 µL) were extracted before the start (prior to the addition of HFO-1123), at 0 h, 1 h, 6 h, and 12 h after addition of HFO-1123. For ^19^F-NMR investigations, aliquots (500 µL) were removed at 0 h and 6 h of the incubation. Incubations were stopped by addition of ice-cold acetonitrile (LC–MS/MS: 300 µL, ^19^F-NMR: 600 µL) to each aliquot and centrifugation at 4 °C and 20,000 × *g* for 15 min. The obtained pellet was resolubilized in acetonitrile (LC–MS/MS: 300 µL, ^19^F-NMR: 600 µL) and again centrifuged (4 °C, 20,000 × *g*, 15 min). The supernatants of both centrifugation steps were combined and the acetonitrile/water phase was evaporated under reduced pressure. The dried residue was reconstituted in 100 µL H_2_0 + 0.1% formic acid (FA) for LC–MS/MS investigations and in 550 µL D_2_O for ^19^F-NMR investigations and stored at − 20 °C until analysis.

#### β-Lyase-mediated biotransformation of 1123-CYS by spectrometric assay and ^19^F-NMR

β-Lyase-mediated cleavage of 1123-CYS was monitored based on the method described by Lash (2007) with the following modifications. Spectrometric studies were carried out with a SpectraMax^®^ Plus 384 Microplate Reader (Molecular Devices, San José, USA) operating at a wavelength of 340 nm. Incubation temperature was set to 37 °C and measurements were performed for 20 min. All measurements were performed in triplicates and in three independent experiments. Per well of a 96-well plate, renal or hepatic cytosolic fractions of rats, mice, minipigs, and rabbits (0.5–2 mg protein/mL) were incubated with NADH/H^+^ (0.1 mM (in K_2_HPO_4_/KH_2_PO_4_ buffer (100 mM, pH 7.4) and 1123-CYS (0.5, 1, 2, 3, 4, 5 mM) in α-keto-γ-(methylthio)-butyrate (KMB) (0.1 mM) in K_2_HPO_4_/KH_2_PO_4_ puffer (100 mM, pH 7.4) and *D*-lactate dehydrogenase (0.1 units/mL) (total volume of 0.1 mL). TCVC, a known substrate for β-lyase-mediated cleavage, was used as a positive control. To confirm β-lyase-dependent cleavage of 1123-CYS, some incubations were performed in the presence of the β-lyase inhibitor AOAA (0.625 mM). To compensate for any NADH oxidation or other nonspecific decrease in absorbance, a blank incubation (absence of cysteine *S*-conjugates) was measured for 20 min. After homogenization, the decrease in the absorbance was measured over 20 min. The decrease in absorbance of the blank incubations was subtracted from the decrease in absorbance of the samples. The amount of pyruvate formed was calculated using a calibration curve, showing linearity over the range of 5 to 50 µM (*R*^2^ values ≥ 0.99).

To study formation of fluorine-containing metabolites, 1123-CYS (5 mM) containing KMB (0.1 mM) was incubated with human, rabbit, and minipig renal and hepatic cytosol (5 mg protein/mL) in a final volume of 1 mL. Incubations were performed for 60 min at 37 °C in a shaking water bath. To monitor β-lyase-dependent cleavage of 1123-CYS, some incubations were performed in the presence of AOAA (5 mM). 0.5 mL aliquots were extracted at 0 and 60 min after the start of the reaction. For sample workup, aliquots were placed on ice and immediately centrifuged at 164,000 × *g* and 4 °C for 30 min. D_2_O was added to the supernatants, yielding a total volume of 0.8 mL.

### Cell culture experiments

Cell viability was determined in proximal tubular cell cultures of pigs (LLC-PK1 cell line), rats (NRK-52E cell line), and humans (HK-2 cell line) exposed to 1123-CYS. HK-2, NRK-52E and LLC-PK1 cells were obtained from the European Collection of Cell Cultures (Porton Down, England) and the American Type Culture Collection (Manassas, USA). Cell lines were cultured in Corning^®^ T75 cell culture flasks (Sarstedt AG, Nürmbrecht, Germany) under standard cell culture conditions (37 °C, 5% CO_2_). HK-2 cells were cultured in Dulbecco’s modified Eagle’s medium (DMEM) and Nutrient Mixture F-12 Ham (Merck Millipore, Darmstadt, Germany) (V:V, 1:1) with 3.15 g/L glucose, 10% fetal bovine serum superior (FBS) (Merck Millipore), 1% penicillin/streptomycin (Merck Millipore), and 2 mM GlutaMAX™-I (Merck Millipore). NRK-52E cells were cultured in DMEM with 4.5 g/L glucose, 10% FBS, 1% penicillin/streptomycin, 2 mM GlutaMAX™-I (ThermoFisher Scientific, Dreieich, Germany), and 1% Gibco™ MEM non-essential amino acid solution (ThermoFisher Scientific). LLC-PK1 cells were cultured in DMEM with 1.0 g/L glucose, 10% FBS, 1% penicillin/streptomycin, 2 mM GlutaMAX™-I, and 25 mM HEPES (Merck Millipore).

To determine cytotoxicity of 1123-CYS, cells were seeded into 96-well plates at a density of 0.01 × 10^6^ cells and allowed to grow to 90–100% confluency for 48 h. Cells were then treated with 1123-CYS at 0, 39.1, 78.1, 156.3, 312.5, 625, 1,250, 2,500, 5,000, and 10,000 µM in the absence or presence of 0.125–0.250 mM AOAA for 48 h under standard cell culture conditions (37 °C, 5% CO_2_). Treatment solutions were prepared by serial dilution of stock solutions of 1123-CYS (10,000 µM) in culture media. After treatment, cell viability was determined using the CellTiter-Glo® Luminescent Cell Viability Assay (Promega, Fitchburg, USA) according to the manufacturer’s instructions. Each assay was performed in three independent experiments carried out in triplicate.

### Statistical analysis

Unless otherwise indicated, Microsoft Excel version 16.66.1 for Mac OS 11.5.2 was used. GraphPad Prism version 9.1.2 (GraphPad Software, San Diego, USA) for Mac OS 11.5.2 was used for analysis of cell culture experiments.

## Results

### GST-dependent biotransformation of HFO-1123 in S9 fractions

**Fig. 2:**
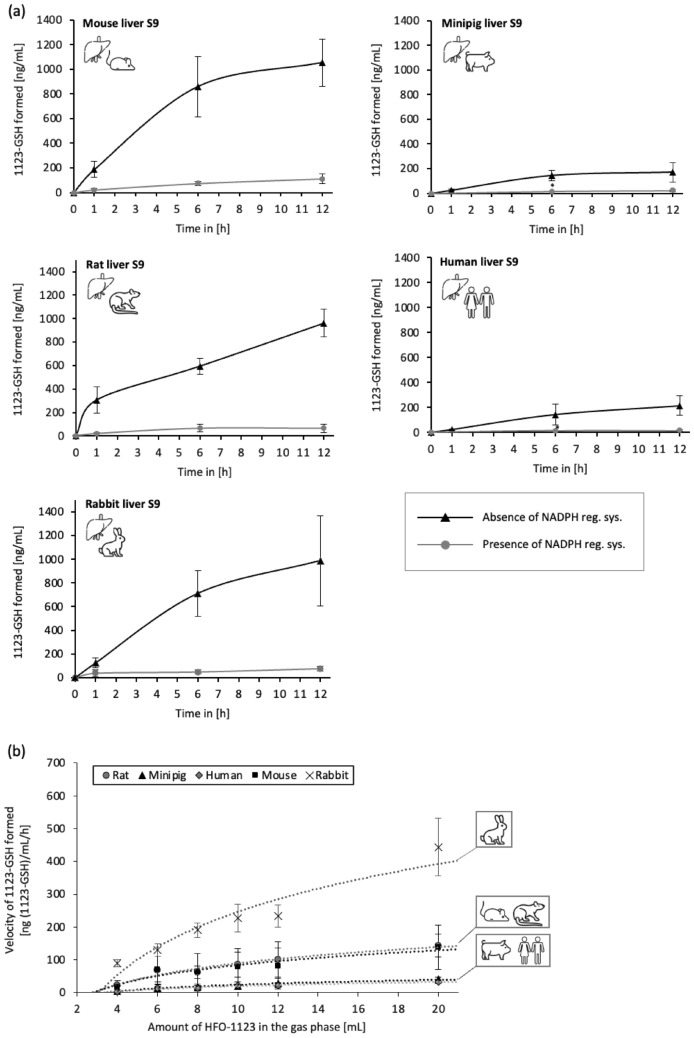
**a** Time-dependent formation of 1123-GSH in hepatic S9 fractions of mice, rats, rabbits, minipigs, and humans in the presence or absence of an NADPH regenerating system. **b** Velocity of 1123-GSH formed (ng 1123-GSH/mL/h) in hepatic S9 fractions of mice, rats, rabbits, minipigs, and humans after incubation with HFO-1123. Results are presented as mean ± standard deviation (*n* = 3)

Time-dependent formation of 1123-GSH was observed in minipig, rabbit, human, rat, and mouse hepatic S9 fractions incubated with HFO-1123 in the presence of GSH. Across species, the rate of 1123-GSH formation was consistently lower in the presence as compared to the absence of the NADPH regenerating system, indicating CYP-450-dependent biotransformation of HFO-1123 in the presence of the NADPH regenerating system (Fig. 2a). In the absence of the NADPH regenerating system, 1123-GSH formation was approximately fivefold higher in hepatic S9 fractions of mouse, rat, and rabbit as compared to human and minipig fractions. These results suggest increased GST-mediated formation of 1123-GSH in rat, mouse, and rabbit compared to human and minipig.

In vitro kinetics of GST-mediated 1123-GSH formation in hepatic S9 fractions incubated with different amounts of HFO-1123 were assessed to characterize enzyme kinetic parameters (K_M_ and V_MAX_) of GST-mediated formation of 1123-GSH. As can be seen from the velocity of 1123-GSH formed plotted against the amount of HFO-1123 in the gas phase (Fig. 2b), markedly higher rates of 1123-GSH formation were detected in rabbit, followed by rat and mouse hepatic S9 fractions. The lowest velocity of 1123-GSH formation was detected in human and minipig hepatic S9 fractions. Due to the relatively low amounts of 1123-GSH formed at low HFO-1123 concentrations in the gas phase, plots to derive K_M_ and V_MAX_ did not, however, show the required linearity to reliably determine K_M_ and V_MAX_.

To check if 1123-GSH is rapidly cleaved via hepatic γ-GT, aminopeptidase, and dipeptidase, 1123-CYS was monitored qualitatively in incubations of HFO-1123 and hepatic S9. 1123-CYS was not detected in incubations with rat and mouse S9, whereas signals corresponding to 1123-CYS were detected in human and minipig hepatic S9, despite the lower rate of 1123-GSH formation. Rabbit hepatic S9 showed slightly higher levels of both 1123-GSH and 1123-CYS compared to other species as judged by signal intensity (Supplementary material, Fig. S4). However, the signals corresponding to 1123-CYS were generally low, suggesting that cleavage of 1123-GSH to 1123-CYS does not occur to a significant extent in hepatic S9. This may suggest that conversion of 1123-GSH to 1123-CYS occurs predominantly in the kidneys, consistent with the higher activity of GSH-processing enzymes in the kidney as compared to the liver (Dekant et al. 1994; Cooper and Hanigan 2018).

Acivicin, an inhibitor of mammalian γ-GT (Gardell and Tate 1980), was further used to evaluate the extent of γ-GT-mediated cleavage of 1123-GSH formed in hepatic S9 fractions. While acivicin had no significant effect on the concentration of 1123-GSH in rat, mouse, human, and minipig hepatic S9 incubated with HFO-1123, an increase in 1123-GSH formation was detected in the presence of acivicin in rabbit hepatic S9 (Supplementary material, Fig. S5). However, as judged by relative signal intensities, γ-GT-mediated cleavage of 1123-GSH was not paralleled by an increase in the concentration of 1123-CYS. To assess if 1123-CYS formed from 1123-GSH is readily cleaved via β-lyase activity, hepatic S9 fractions were incubated with HFO-1123 in the presence and absence of aminooxyacetic acid (AOAA). Only rabbit S9 showed a notable increase in 1123-CYS signal intensity in the presence of AOAA (S/N-ratio: 12/1) as compared to the absence of AOAA (S/N-ratio: 3/1), indicating AOAA-mediated inhibition of β-lyase cleavage of 1123-CYS (for LC–MS/MS chromatograms, see supplementary materials, Fig. S6). However, as the intensities of signals assigned to 1123-CYS around the limit of detection in all hepatic S9, hepatic γ-GT, aminopeptidase and dipeptidase do not appear to contribute significantly to the cleavage of 1123-GSH.

**Fig. 3.**
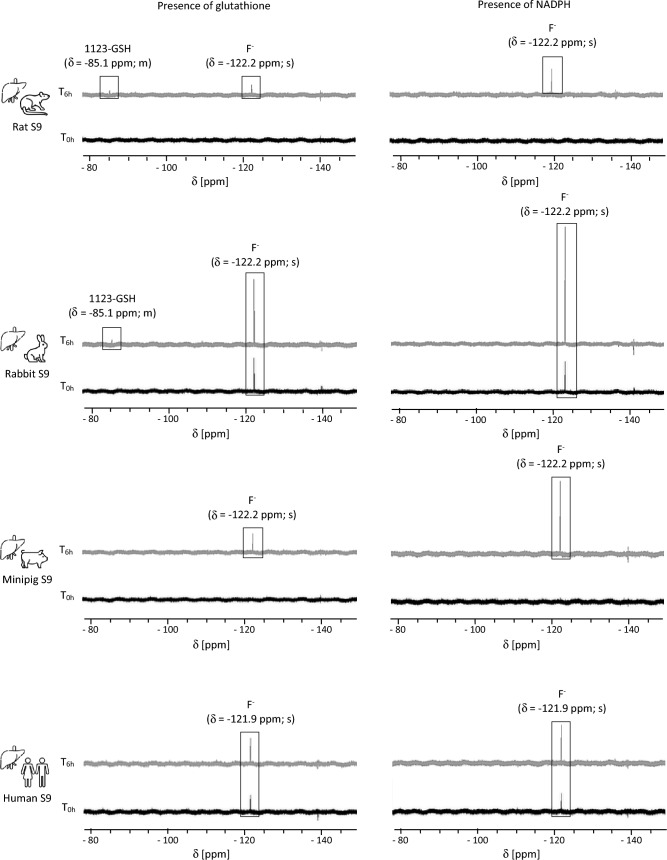
^19^F-NMR spectra of rat, human, minipig, and rabbit hepatic S9 subcellular fractions incubated with HFO-1123 in the presence of either GSH or the NADPH regenerating system. The black spectra show fluorine-containing metabolites present at the beginning of the incubation (T_0h_) and the gray spectra reveal fluorine-containing metabolites after 6 h of incubation (T_6h_). ^19^F-NMR signals corresponding to 1123-GSH (δ = − 85.1 ppm, m) were detected in hepatic rat and rabbit S9 fractions in the presence of GSH, but not in S9 of other species. Compared to human and rat S9, increased F^−^ formation was detected in minipig and rabbit hepatic S9 in the presence of an NADPH regenerating system. In contrast to the synthesized 1123-GSH reference compound, which is characterized by two signals (δ = − 85.0 ppm, m, 2F; δ = − 227.9 ppm, m, 1F), only the signal corresponding to the C_1_ bound fluorine atoms (δ = − 85.1 ppm, m, 2F) was detected in rat and rabbit incubations close to the limit of detection. Absence of the additional signal (δ = − 227.9 ppm, m, 1F) and signals in other S9 fractions can be explained by the lower sensitivity of ^19^F-NMR compared to LC–MS/MS

While 1123-GSH is expected to be the major metabolite formed by GST-mediated metabolism of HFO-1123, it is also possible that other potentially toxic metabolites may be formed by GST-mediated metabolism. To identify potential further metabolites, hepatic S9 fractions incubated with HFO-1123 in the presence or absence of GSH or an NADPH regenerating system were analyzed by ^19^F-NMR. Consistent with LC–MS/MS analysis showing increased 1123-GSH formation in rat and rabbit hepatic S9 compared to S9 of other species, ^19^F-NMR-signal corresponding to 1123-GSH (δ = − 85.1 ppm, m) was detected only in rat and rabbit hepatic S9 fractions in the presence of GSH (Fig. 3). Due to the lower sensitivity of ^19^F-NMR as compared to LC–MS/MS, only the signal corresponding to the C_1_ bound fluorine atoms was detected in the S9 incubations, with signal intensities close to the limit of detection. In addition to 1123-GSH, time-dependent formation of F^−^ was detected in hepatic S9 fractions, and this was further enhanced in the presence of the NADPH regenerating system. Based on signal intensities, highest amounts of F^−^ were formed in minipig and rabbit S9 compared to the rat, mouse, and human S9, suggesting a higher rate of CYP-450-dependent metabolism of HFO-1123 in species most susceptible to HFO-1123 toxicity. Fluorine-containing metabolites other than F^−^ and 1123-GSH were not detected in hepatic S9 fractions incubated with HFO-1123 using ^19^F-NMR analysis.

Since renal tissue also exhibits GST and γ-GT activity (reviewed by Hanna and Anders (2019)), we considered that GSH-dependent biotransformation of HFO-1123 to 1123-GSH and 1123-CYS in the kidney may contribute to the species-differences in HFO-1123 toxicity. Time-dependent formation of 1123-GSH and 1123-CYS was observed in incubation of HFO-1123 with human, minipig, and rat kidney S9 (data not shown). However, consistent with the lower GST activity but higher γ-GT activity in kidney as compared to liver, the amount of 1123-GSH in minipig and human kidney S9 was approximately tenfold and approximately 100-fold lower in rat kidney S9 (compared to liver S9), whereas 1123-CYS was formed at comparable levels in renal and hepatic fractions as judged by peak area.

### β-Lyase-mediated biotransformation of 1123-CYS in cytosolic fractions

**Fig. 4:**
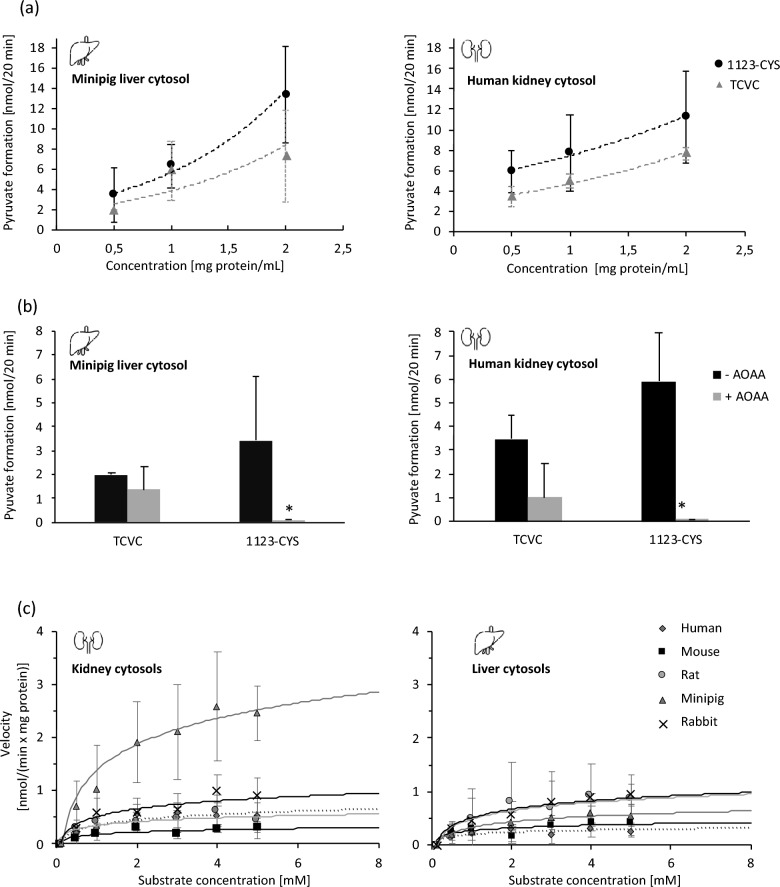
**a** Protein concentration-dependent formation of pyruvate in incubations of minipig liver and human kidney cytosol with 1123-CYS and **b** inhibition of pyruvate formation in incubations of cytosols and 1123-CYS/TCVC in the presence of AOAA. **c** In vitro kinetics of β-lyase-mediated cleavage of 1123-CYS in renal and hepatic cytosols (Mouse, rat, minipig, rabbit, and human). Data are presented as mean ± standard deviation of three independent experiments performed in triplicates (*n* = 3)

To test if 1123-CYS undergoes β-lyase-mediated cleavage, leading to the formation of MFA in vitro, renal and hepatic cytosolic fractions were incubated with 1123-CYS. Time- and protein concentration-dependent formation of pyruvate was observed in cytosolic fractions incubated with 1123-CYS and TCVC (Fig. 4a). TCVC is a known substrate of β-lyase and was used as a positive control. The β-lyase inhibitor AOAA completely blocked pyruvate formation in cytosolic fractions incubated with 1123-CYS, further confirming 1123-CYS as a substrate of β-lyases (Fig. 4b). Enzyme kinetics of β-lyase-mediated cleavage of 1123-CYS revealed increased β-lyase activity in renal minipig cytosol as compared to liver and renal cytosolic fractions of other species. β-Lyase activity in renal rabbit cytosol was slightly increased compared to renal cytosol of human, rat, and mouse but lower than renal minipig cytosol (Fig. 4c; Table 1).

**Table 1:** Calculated values for V_max_ and K_m_ obtained with cytosolic fractions by Eadie–Hofstee Plot and Lineweaver–Burk Plot (Lineweaver and Burk 1934; Eadie 1942)

Cytosolic fraction		Eadie–Hofstee		Lineweaver–Burk	
		V_max_ [nmol (pyruvate formed)/(mg protein x min)]	K_m_ [mM]	V_max_ [nmol (pyruvate formed)/(mg protein x min)]	K_m_ [mM]
Human	Kidney	0.77 ± 0.26	1.22 ± 0.42	0.83 ± 0.29	1.50 ± 0.53
	Liver	0.31 ± 0.05	0.30 ± 0.05	0.29 ± 0.05	0.27 ± 0.04
Mouse	Kidney	0.29 ± 0.10	0.25 ± 0.08	0.33 ± 0.1	0.62 ± 0.2
	Liver	0.38 ± 0.17	0.17 ± 0.07	0.35 ± 0.16	0.16 ± 0.07
Rat	Kidney	0.55 ± 0.24	0.35 ± 0.19	0.55 ± 0.23	0.36 ± 0.2
	Liver	1.04 ± 0.63	0.94 ± 0.57	1.14 ± 0.69	1.13 ± 0.68
Minipig	Kidney	3.63 ± 1.35	2.06 ± 0.77	3.55 ± 1.33	2.03 ± 0.76
	Liver	0.74 ± 0.27	1.40 ± 0.51	0.71 ± 0.25	1.32 ± 0.48
Rabbit	Kidney	1.04 ± 0.15	1.01 ± 0.37	1.14 ± 0.14	0.83 ± 0.42
	Liver	1.12 ± 0.10	1.29 ± 0.27	1.06 ± 0.23	1.15 ± 0.17

**Fig. 5:**
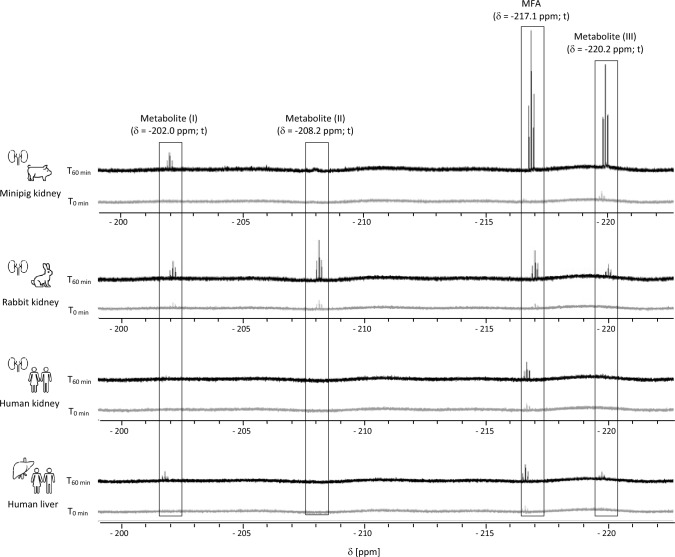
Identification of species-differences in β-lyase-dependent formation of MFA and metabolites containing a CH_2_F unit in cytosolic fractions of minipigs, rabbits and humans. Resonances were assigned to MFA (δ = − 217.1 ppm; t, *J*_HF_ = 48.4 Hz); unknown CH_2_F unit-containing metabolite (I) (δ = − 202.0 ppm; t, *J*_HF_ = 48.5 Hz); unknown CH_2_F unit-containing metabolite (II) ((δ = − 208.2 ppm, t, *J*_*HF*_ = 48.5 Hz); unknown CH_2_F unit-containing metabolite (III) (δ = − 220.2 ppm; t, *J*_*HF*_ = 48.5 Hz)

**Fig. 6:**
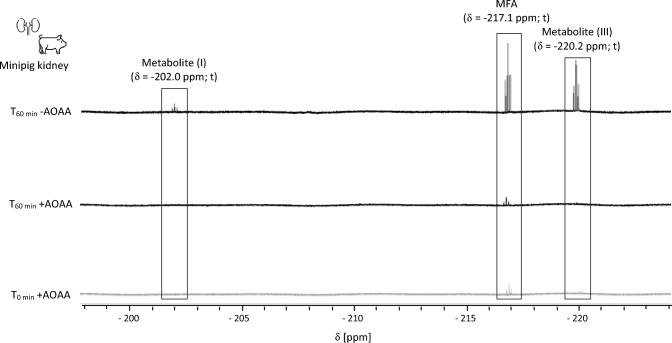
Inhibition of the formation of MFA and unknown metabolites containing a CH_2_F unit in the presence of AOAA in renal minipig cytosol. Resonances were assigned MFA (δ = − 217.1 ppm; t, *J*_HF_ = 48.4 Hz); unknown CH_2_F unit-containing metabolite (I) (δ = − 202.0 ppm; t, *J*_HF_ = 48.5 Hz) and unknown CH_2_F unit-containing metabolite (III) (δ = − 220.2 ppm; t, *J*_*HF*_ = 48.5 Hz)

As β-lyase activity towards 1123-CYS was observed in cytosols of all species, with increased activity in renal minipig cytosols, ^19^F-NMR was used to identify F-containing metabolites formed from β-lyase cleavage of 1123-CYS. ^19^F-NMR revealed the formation of signals assigned to F^−^ and MFA in renal minipig cytosol (Fig. 5). Formation of MFA and F^−^ was blocked in the presence of AOAA, confirming β-lyase-dependent formation (Fig. 6). In addition to MFA and F^−^, unknown metabolites containing an FCH_2_ unit were detected in incubations of renal minipig, but their exact structure could not be identified. MFA, F^−^, the unknown metabolites, and an additional unknown metabolite containing an FCH_2_ unit were also identified in rabbit cytosol incubated with 1123-CYS (Fig. 5). Consistent with the lower β-lyase activity in rabbit cytosol compared to renal minipig cytosol, the amounts of F^−^, MFA, and unknown metabolites formed were lower in renal and hepatic rabbit cytosol compared to renal minipig cytosol. Incubations of human kidney cytosol with 1123-CYS showed a minimal, but time-dependent formation of signals assigned to F^−^ and MFA. However, the intensity of the MFA signal obtained in renal minipig cytosol was approximately sevenfold higher as compared to the MFA signal in renal and hepatic human cytosols (Fig. 5). The unknown metabolites were not detected in renal human fractions and only at trace levels in hepatic human fractions.

### Cytotoxicity of 1123-CYS

**Fig. 7:**
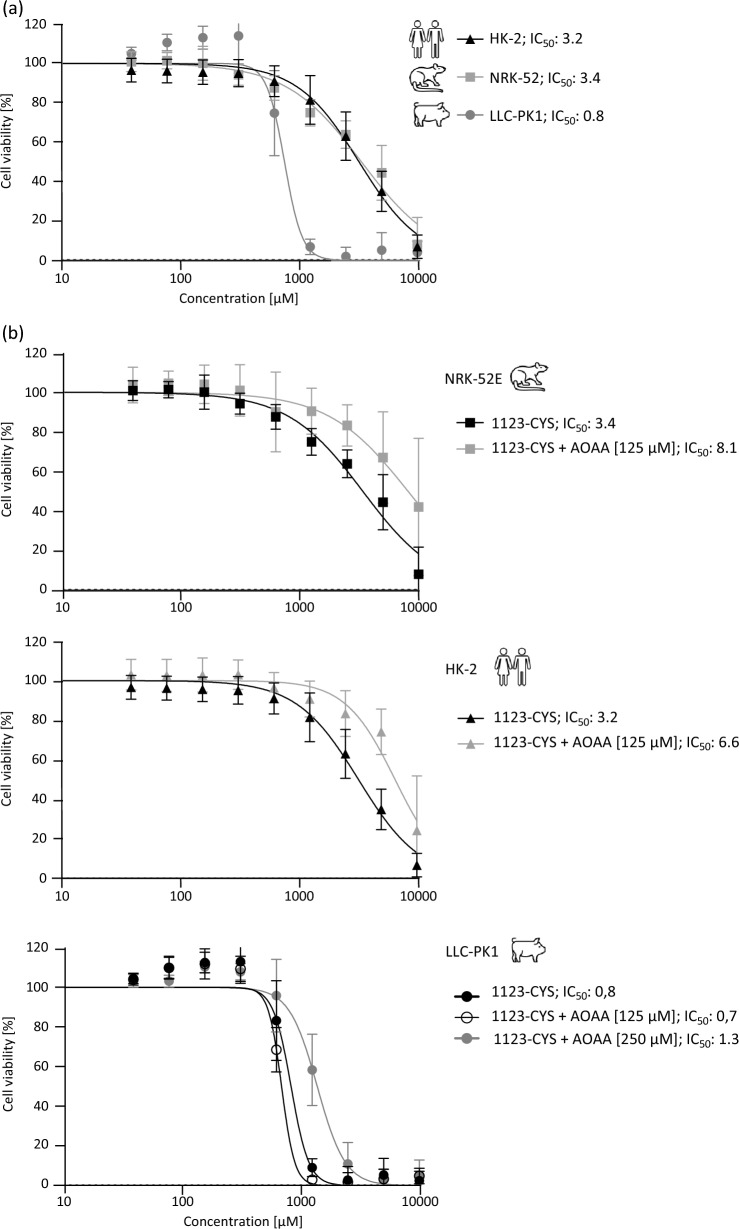
Cytotoxicity of 1123-CYS in human (HK-2), rat (NRK-52E) and porcine (LLC-PK1) proximal tubular cell lines after 48 h treatment with **a** 1123-CYS and **b** 1123-CYS in the absence and presence of the β-lyase inhibitor AOAA (NRK-52E and HK-2: 125 µM AOAA; LLC-PK1: 125 µM/250 µM AOAA). Results are presented as mean ± standard deviation of three independent experiments, each performed in triplicates

To further confirm the role of β-lyases in 1123-CYS toxicity, cytotoxicity of 1123-CYS was comparatively assessed in HK-2 (human), NRK-52E (rat), and LLC-PK1 (pig) proximal tubular cell lines in the absence and presence of the β-lyase inhibitor AOAA. Interestingly, LLC-PK1 cells were found to be more susceptible to 1123-CYS cytotoxicity as compared to NRK-52E and HK-2 cells (Fig. 7). The β-lyase inhibitor AOAA ameliorated 1123-CYS toxicity in all proximal tubular cell lines, indicating that cytotoxicity of 1123-CYS depends at least to some extent on β-lyase enzyme activity (Fig. 7). Higher concentrations of AOAA were needed to produce a protective effect in LLC-PK1 cells (250 µM) as compared to NRK-52E and HK-2 cells (125 µM), presumably due to the higher β-lyase activity of porcine kidney as compared to that of other species.

## Discussion

Species-differences in HFO-1123 biotransformation are considered to contribute to the species-differences in HFO-1123 toxicity observed in experimental animals. It is thought that GSH-dependent biotransformation via glutathione-S-transferases (GST) to 1123-GSH, primarily in the liver, and subsequent β-lyase-mediated cleavage of the corresponding cysteine conjugate (1123-CYS) predominantly in the kidney may play a key role in the bioactivation and toxicity of HFO-1123. Cleavage of 1123-CYS via β-lyases, which are abundantly expressed in kidneys (Cooper and Pinto 2006), is anticipated to give rise to MFA and is therefore assumed to be the critical step in the toxicity of HFO-1123 in sensitive species. The aim of the present work was to identify potential species-differences in the in vitro biotransformation of HFO-1123 via the mercapturic acid pathway in susceptible vs. less susceptible species and humans.

Toxicity of halocarbons is generally based on CYP-450-dependent and/or mercapturic acid pathway (MAP)-associated biotransformation, leading to several metabolites responsible for in vitro an in vivo toxicity. Based on the individual halocarbon, the contribution of each pathway to bioactivation differs significantly. For instance, bioactivation of trichloroethene (TRI) and vinyl chloride are predominantly based on CYP-450-dependent activation (Kapp 2014; Lash et al. 2014), whereas tetrafluoroethylene, hexachloro-1,3-butadiene, and perfluoropropene are generally activated via the mercapturic acid pathway (Cooper and Hanigan 2010). However, minor biotransformation pathways can contribute significantly to overall toxicity and especially to organ-specific toxicity, as evidenced by TRI (Lash et al. 2000). Depending on the respective haloalkene, species- and sex-differences in GST activity are reported that may be partially linked to specific toxicity of the formed glutathione conjugate (Lash et al. 1998, 1999). Moreover, species-differences in β-lyase activity are described. Up to 11 enzymes are suggested to be involved in the β-cleavage of cysteine S-conjugates, showing substrate-, tissue-, species-, sex-, and interindividual-specific activity towards cysteine S-conjugates derived from halocarbons (Cooper and Pinto 2006). For example, rats show a three-to-tenfold increase in β-lyase-dependent cleavage of S-(1,2-dichlorovinyl)-L-cysteine (the cysteine S-conjugate of TRI) compared to an analogous human kidney model (McGoldrick et al. 2003). Also, interindividual variations in β-lyase activity towards DCVC are described that suggest a genetic polymorphism exists for β-lyases (McCarthy et al. 1994). It can therefore be anticipated that biotransformation of HFO-1123, especially β-lyase activity towards 1123-CYS, could differ significantly between species and individuals and may explain the susceptibility of minipigs and rabbits towards HFO-1123.

While 1123-GSH formation as the initial step in the biotransformation of HFO-1123 was increased in liver S9 of rat, mouse, and rabbit compared to human and minipig, higher rates of β-lyase-mediated cleavage of 1123-CYS were observed in renal cytosol of minipigs as compared to cytosol from other species (rabbits > rats ~ humans > mice). Analysis by ^19^F-NMR confirmed time-dependent formation of MFA in renal cytosol of minipigs and to a lesser extent in rabbits, providing support for the hypothesis that enzymatic cleavage by β-lyases may account for the toxicity of HFO-1123 in susceptible species.

Results from this work clearly establish HFO-1123 as a substrate for GST-mediated biotransformation, leading to formation of 1123-GSH in hepatic and to a lesser extent in renal S9 fractions, in line with an increased GST expression in the liver compared to the kidney (Hanna and Anders 2019). Besides 1123-GSH and F^−^, which is presumed to result from CYP-450-dependent metabolism of HFO-1123, no further metabolites were detected in hepatic S9 by ^19^F-NMR, confirming 1123-GSH as the major metabolite derived from GST-dependent HFO-1123 metabolism. 1123-CYS was detected at low levels only by LC–MS/MS, indicating that γ-GT/aminopeptidase (APN)/dipeptidase (DPP)-dependent processing of 1123-GSH to its corresponding cysteine S-conjugate does not occur at significant rates in liver S9. Species-differences in the rate of GST-mediated biotransformation of HFO-1123 were evident, with increased GST activity towards HFO-1123 in rat, mouse, and rabbit compared to human and minipig hepatic S9.

Across all species, considerably lower amounts of 1123-GSH were detected in the presence of an NADPH regenerating system, indicating CYP-450 mediated biotransformation as an additional route of HFO-1123 metabolism. CYP-450-dependent metabolism appeared to occur at higher rates in minipig and rabbit hepatic S9, as evidenced by increased signals corresponding to F^−^ in ^19^F-NMR spectra in the presence of an NADPH regenerating system. Other fluorine-containing metabolites, such as *S*-(1,1,2-trifluoro-2-hydroxyethyl)-*L*-glutathione that might be expected to be formed by reaction of the CYP-450-dependent intermediate epoxide with GSH, were not detected in the presence of the NADPH regenerating system. This suggests hydrolysis of the intermediate epoxide, giving rise to F^−^ and oxalic acid, as the predominant route of biotransformation of the intermediate epoxide, whereas GST-dependent processing of this CYP-450-dependent metabolite appears to occur only to a minor extent. As stable metabolites with selective toxicity [as compared to CYP-450-dependent biotransformation of TRI (Lash et al. 2014)] were not identified in the presence of the NADPH regenerating system, it may be assumed that this pathway does not directly lead to bioactivation of HFO-1123.

Since increased GST-mediated biotransformation of HFO-1123 in rabbit, rat, and mouse compared to human and minipig hepatic S9 is not entirely consistent with the hypothesis of increased biotransformation of HFO-1123 in minipigs and rabbits being causal for in vivo toxicity, differences in the further metabolic fate of 1123-GSH in the presence of hepatic S9 were investigated. LC–MS/MS analysis indicated higher rates of cleavage of 1123-GSH to 1123-CYS in rabbit, human, and minipig compared to rat and mouse hepatic S9, although overall the levels of 1123-CYS found were low. Additionally, acivicin blocked cleavage of 1123-GSH significantly in rabbit hepatic S9 fractions, suggesting that γ-GT-dependent processing of 1123-GSH occurs at higher rates in rabbit hepatic S9 as compared to liver S9 of other species. However, inhibition of γ-GT by acivicin was not paralleled by changes in levels of 1123-CYS formed.

Analysis of β-lyase-mediated biotransformation of 1123-CYS in hepatic and renal cytosolic fractions confirmed 1123-CYS as a substrate of β-lyase cleavage. This was supported by time- and protein-dependent formation of pyruvate and MFA and inhibition of their formation in the presence of the specific β-lyase inhibitor AOAA. In liver cytosols, only minor differences in β-lyase activity towards 1123-CYS were evident between species. However, β-lyase activity and β-lyase-dependent formation of MFA were markedly higher in minipig renal cytosol compared to hepatic and renal cytosolic fractions of other species (NZW rabbits > rats ~ humans > mice). These in vitro biotransformation data, combined with the protective effects of the β-lyase inhibitor on 1123-CYS cytotoxicity in renal epithelial cells, provide experimental support for β-lyase-dependent cleavage of 1123-CYS to MFA as a critical step in HFO-1123 toxicity.

A key role of MFA in HFO-1123 toxicity is also consistent with the biochemical changes observed in minipigs exposed to HFO-1123 via inhalation, which were characterized by a concentration-dependent increase in plasma citrate levels (85–200 ppm HFO-1123) accompanied by excretion of MFA in urine of minipigs administered 200 ppm of HFO-1123 for 4 h (Wako 2019a). In contrast, no toxicity or significant changes in plasma citrate levels were found in rats exposed to HFO-1123 (> 20.000 ppm) (Wako 2019b). Additionally, significantly lower amounts of MFA formed in human cytosolic fractions as compared to minipig fractions in the present study are in line with the detection of MFA in urine but absence of toxicity in chimeric mice with a humanized liver exposed to HFO-1123 (Wako 2018a). In line with elevated plasma citrate levels in minipigs exposed to HFO-1123 that can be attributed to the interaction of MFA with aconitase in the citric acid cycle, increased concentrations of citrate in plasma and cardiac tissue were noticed with 1-fluoro-2-haloalkanes, which are metabolized to fluoroacetate in rats, causing mortality and fluoroacetate-related signs of toxicity (convulsion, apathy, and hunched posture) (Keller et al. 1996). Increased plasma citrate levels in minipigs exposed to HFO-1123 associated with excretion of MFA are in line with the increased β-lyase activity detected in the susceptible species in the present study and may explain the mortality and toxicity in minipigs exposed to HFO-1123.

Besides MFA and F^−^, β-lyase-dependent formation of unknown metabolites containing an FCH_2_ unit was observed predominantly in incubations of 1123-CYS with cytosolic fractions of minipig and rabbit, i.e., species susceptible to HFO-1123 toxicity. Unfortunately, complete structural identification of these metabolites was not possible. However, chemical shift and multiplicity suggest a structural similarity to MFA. Considering that these metabolites are linked to β-lyase-mediated cleavage of 1123-CYS to MFA, it is possible that these represent MFA conjugates with amino acids (e.g., glycine), acetyl-CoA-thioesters or glucuronides, that can be expected as MFA-derived metabolites. Taken together, our data suggest a critical role of β-lyase-mediated cleavage of 1123-CYS to MFA and structurally related metabolites in HFO-1123 toxicity in susceptible animal species. Based on the available data, biotransformation of HFO-1123 via the mercapturic acid pathway may be expected to occur not only in sensitive species but also in humans, albeit at much lower rate.

Interestingly, porcine kidney cells were also more susceptible to 1123-CYS cytotoxicity as compared to human (HK-2) and rat (NRK-52E) kidney cells. Also, higher concentrations of the β-lyase inhibitor were required to elicit a protective effect against 1123-CYS toxicity in the porcine cell line as compared to rat and human proximal tubular cells. This may reflect higher β-lyase activity and associated increased MFA formation in the porcine cell line. However, whether the differences in sensitivity to 1123-CYS toxicity between these cell lines truly reflect the species-differences observed in HFO-1123 toxicity in vivo or rather differences in the background, physiological state, and differentiation status, including altered expression of transporters and xenobiotic metabolizing enzymes in the immortalized cells remains to be established.

## Conclusion

In summary, in vivo and in vitro data suggest an important role of the mercapturic acid pathway in the bioactivation of HFO-1123. In particular, the extent of β-lyase-mediated cleavage of 1123-CYS leading to MFA in the kidney, an organ rich in β-lyases, may play a critical part in the mechanism of toxicity observed in susceptible animals. The significantly increased rates of β-lyase-mediated cleavage of 1123-CYS and associated formation of MFA as the ultimate toxicant in minipigs compared to rodent and human cytosolic fractions are in line with the unique sensitivity of the minipig to HFO-1123 toxicity observed in in vivo studies and may suggest that toxicity data obtained from minipigs may over-predict HFO-1123 toxicity to humans. Liver metabolism of HFO-1123 to 1123-GSH as the initial step in the mercapturic acid pathway was, however, lower in the susceptible species (minipigs and rabbits) compared to the less susceptible species (mouse and rat). Thus, the available data do not allow to draw firm conclusions regarding the relative contribution of the mercapturic acid pathway to HFO-1123 biotransformation in these species and particular in humans. It is conceivable that other organs (e.g., lungs, respiratory epithelium) may also contribute to GSH-dependent biotransformation of HFO-1123 (Forkert et al. 2005, 2006). To this end, investigation of the in vivo metabolism of HFO-1123 in susceptible vs. less susceptible species may provide a better understanding of the causes underlying the species-differences in HFO-1123 toxicity. Such experiments may then allow to draw conclusions on the safety of HFO-1123 in humans.

### Supplementary Information

Supplementary file1 Figure S. 1: 19F-NMR (A.) spectra and {19F-19F}-correlation NMR (B.) of 1123-GSH after purification; Figure S. 2: 1H-NMR (A.), 13C-NMR (B.) and {13C-1H}-correlation NMR (C.) spectra of 1123-GSH after purification; Figure S. 3: 19F-NMR spectra of purified 1123-CYS; Figure S. 4: LC-MS/MS chromatograms obtained from incubations of HFO-1123 and hepatic S9 fractions of mice, rats, rabbits, minipigs and humans showing signals corresponding to 1123-GSH; Figure S. 5: Time-depended formation of 1123-GSH in hepatic S9 fractions of mice, rats, rabbits, minipigs and humans in the presence or absence acivicin; Figure S. 6: LC-MS/MS chromatograms obtained from incubations of HFO-1123 and hepatic S9 fractions of mice, rats, rabbits, minipigs and humans showing signals corresponding to 1123-CYS in the presence or absence of the β-lyase inhibitor AOAA.

## Data Availability

All data supporting the findings of this study are available within the paper and its Supplementary Information. Raw data are available from the corresponding author upon reasonable request.

## Abbreviations

β-Lyase: Cysteine *S*-conjugate β -lyase
γ-GT: γ-Glutamyltransferase
1123-CYS: *S*-(1,1,2-Trifluoroethyl)-*L*-cysteine
1123-GSH: *S*-(1,1,2-Trifluoroethyl)-*L*-glutathione
AOAA: Amiooxyacetic acid
APN: Aminopeptidase n
DCVC: *S*-(1,2-Dichlorovinyl)-*l*-cysteine
DCVG: *S*-(1,2-Dichlorovinyl)-*l*-glutathione
GSH: Glutathione
GST: Glutathione *S*-transferase
MAP: Mercapturic acid pathway
MFA: Monofluoroacetic acid
minipig: Goettingen^®^ minipig
mouse: CD1 mouse
rabbit: New Zealand white rabbit
rat: Sprague–Dawley^®^ rat
TRI: Trichloroethene
