# Correction: Species-differences in the in vitro biotransformation of trifluoroethene (HFO-1123)

**DOI:** 10.1007/s00204-023-03640-y

**Published:** 2023-12-06

**Authors:** R. Dekant, R. Bertermann, J. Serban, S. Sharma, M. Shinohara, Y. Morizawa, H. Okamoto, W. Brock, W. Dekant, A. Mally

**Affiliations:** 1 Department of Toxicology, University of Würzburg, Versbacher Strasse 9, 97078 Würzburg, Germany; 2 Department of Inorganic Chemistry, University of Würzburg, Am Hubland, 97074 Würzburg, Germany; 3 Chemicals Company, AGC Inc, CSR Office, 1-5-1, Marunouchi, Chiyoda-ku, Tokyo, 100-8405 Japan; 4 Brook Scientific Consulting LLC, Hilton Head Island, SC USA

**Correction: Archives of Toxicology (2023) 97:3095–3111** 10.1007/s00204-023-03603-3

The original version of this article unfortunately contained a mistake. The presentation of Fig. 5 was incorrect. The corrected Fig. 5 is given below.

**Fig. 5:**
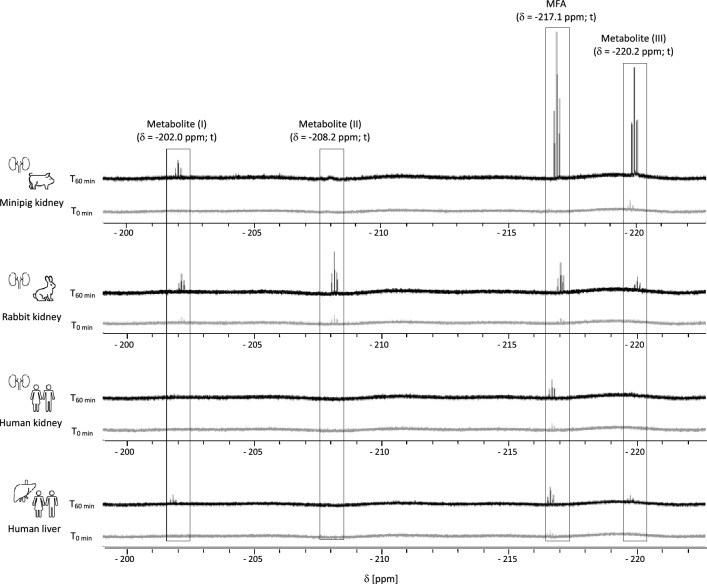
Identification of species-differences in β-lyase dependent formation of MFA and metabolites containing a CH_2_F unit in cytosolic fractions of minipigs, rabbits and humans. Resonances were assigned to MFA (*δ* = − 217.1 ppm; *t*, *J*_HF_ = 48.4 Hz); unknown CH_2_F unit containing metabolite (I) (*δ* = − 202.0 ppm; *t*, *J*_HF_ = 48.5 Hz); unknown CH_2_F unit containing metabolite (II) (*δ* = − 208.2 ppm, *t*, *J*_HF_ = 48.5 Hz); unknown CH_2_F unit containing metabolite (III) (*δ* = − 220.2 ppm; *t*, *J*_HF_ = 48.5 Hz)

