# Supplementary material for: Species-differences in the in vitro biotransformation of trifluoroethene (HFO-1123)
Source: Arch Toxicol. 2023 Oct 4;97(12):3095–111. doi: 10.1007/s00204-023-03603-3 (PMC10567879; doi:10.1007/s00204-023-03603-3)
Supplement: Supplementary file 1 — Supplementary file1 Figure S. 1: 19F-NMR (A.) spectra and {19F-19F}-correlation NMR (B.) of 1123-GSH after purification; Figure S. 2: 1H-NMR (A.), 13C-NMR (B.) and {13C-1H}-correlation NMR (C.) spectra of 1123-GSH after purification; Figure S. 3: 19F-NMR spectra of purified 1123-CYS; Figure S. 4: LC-MS/MS chromatograms obtained from incubations of HFO-1123 and hepatic S9 fractions of mice, rats, rabbits, minipigs and humans showing signals corresponding to 1123-GSH; Figure S. 5: Time-depended formation of 1123-GSH in hepatic S9 fractions of mice, rats, rabbits, minipigs and humans in the presence or absence acivicin; Figure S. 6: LC-MS/MS chromatograms obtained from incubations of HFO-1123 and hepatic S9 fractions of mice, rats, rabbits, minipigs and humans showing signals corresponding to 1123-CYS in the presence or absence of the β-lyase inhibitor AOAA. [file 204_2023_3603_MOESM1_ESM.docx]

**Supplementary material**

**Figure S.1**


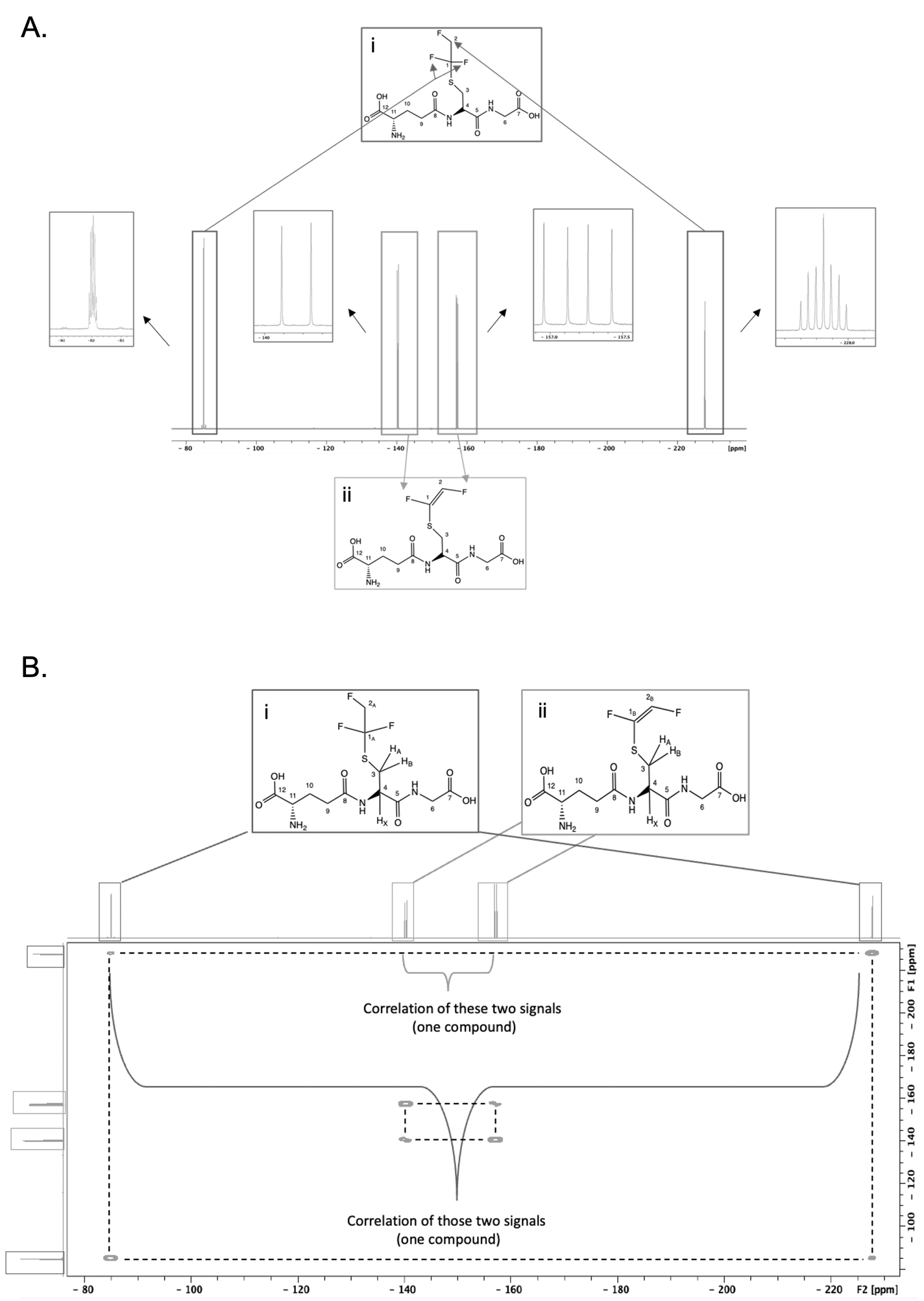


Figure S. 1 ^19^F-NMR (A.) spectra and {^19^F-^19^F}-correlation NMR (B.) of 1123-GSH (i) after purification, revealing *trans*-(1,2-difluoro-ethylen)-*L*-glutathione (ii) as a side product of synthesis.

**Figure S.2**


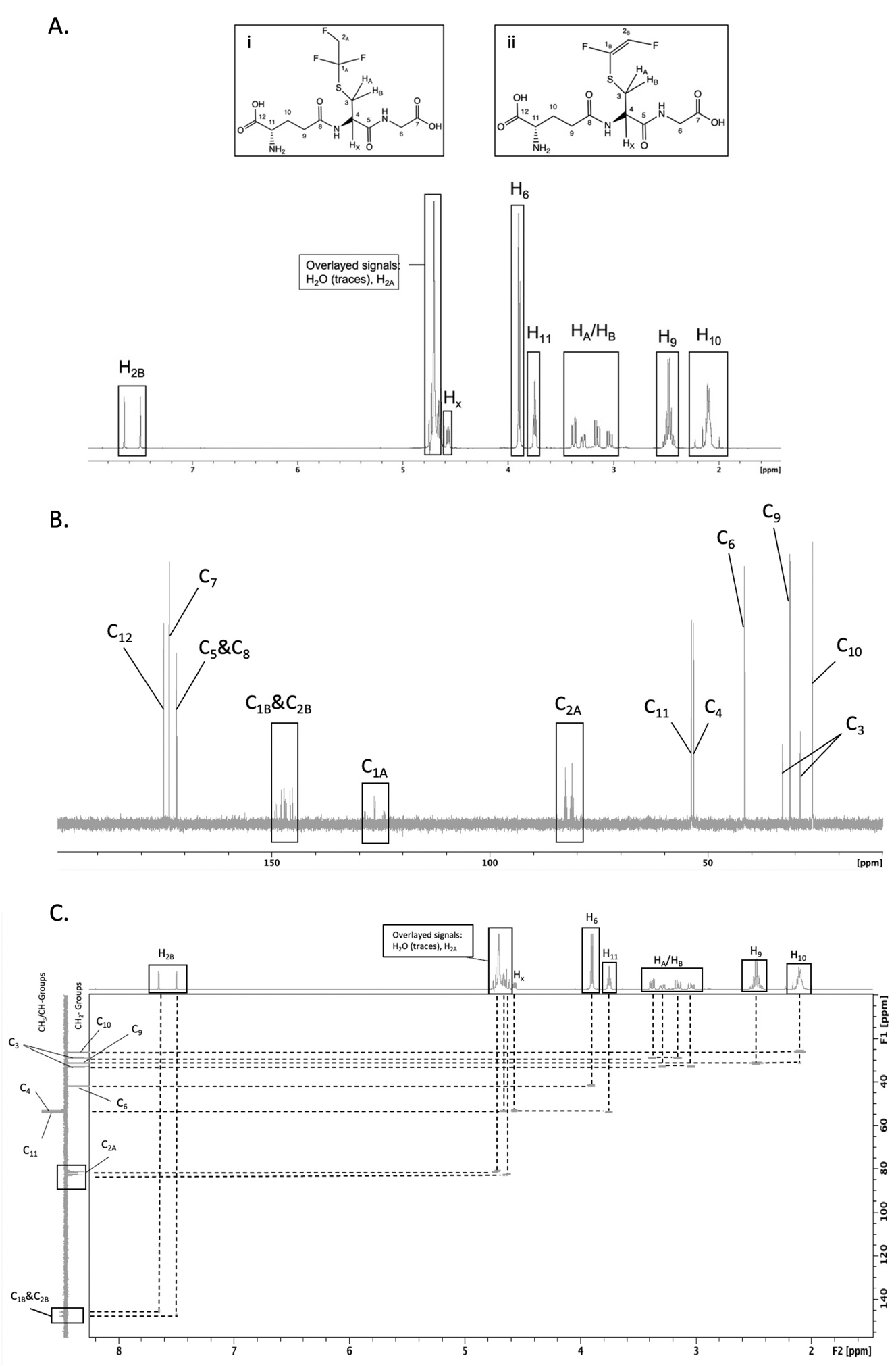


Figure S. 2 ^1^H-NMR (A.), ^13^C-NMR (B.) and {^13^C-^1^H}-correlation NMR (C.) spectra of 1123-GSH (i) after purification, revealing *trans*-(1,2-difluoro-ethylen)-*L*-glutathione (ii) as a side product of synthesis.

**Figure S.3**


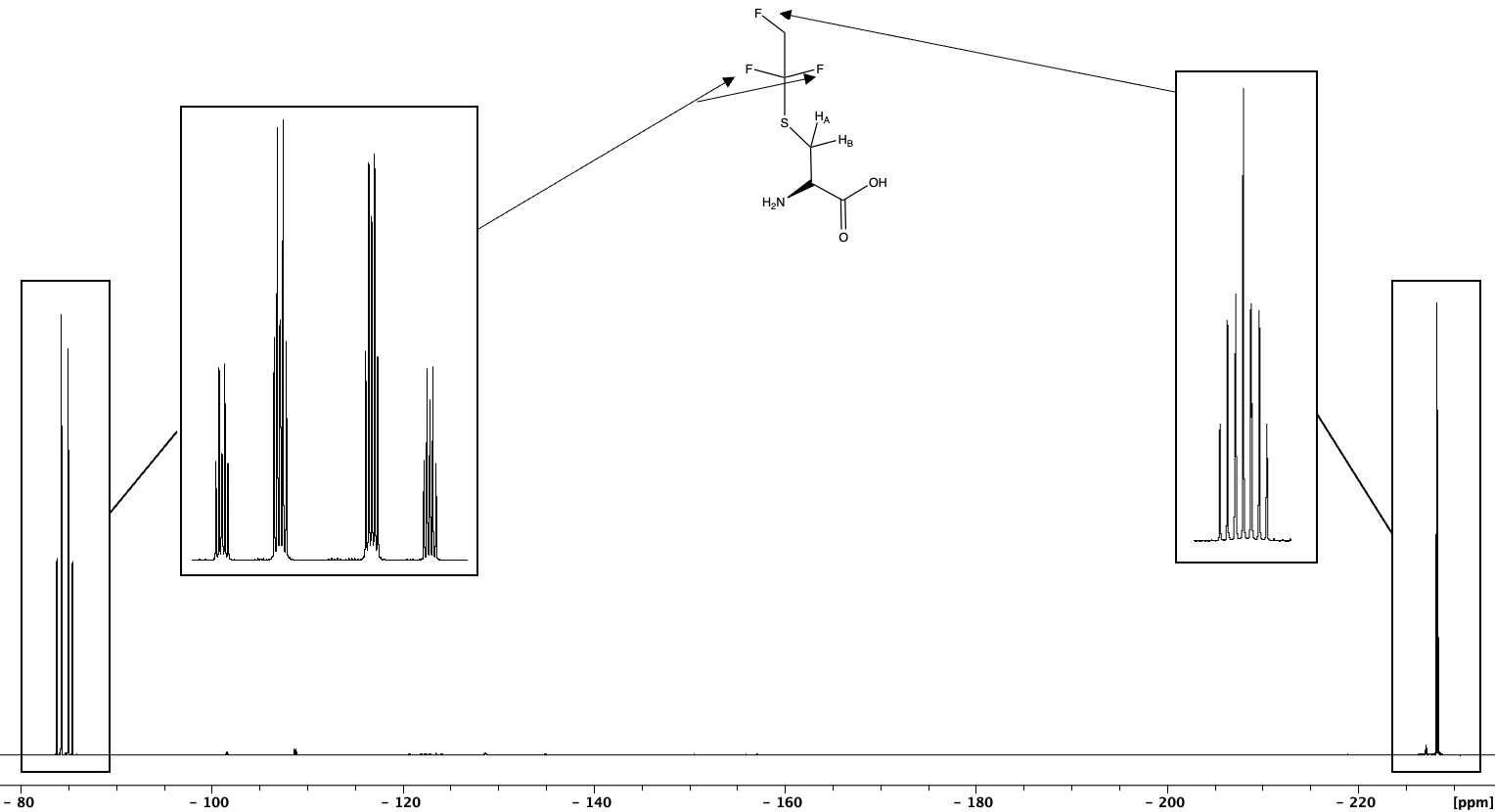


**Figure S. 3** ^19^F-NMR spectra of purified 1123-CYS.

**Figure S.4**


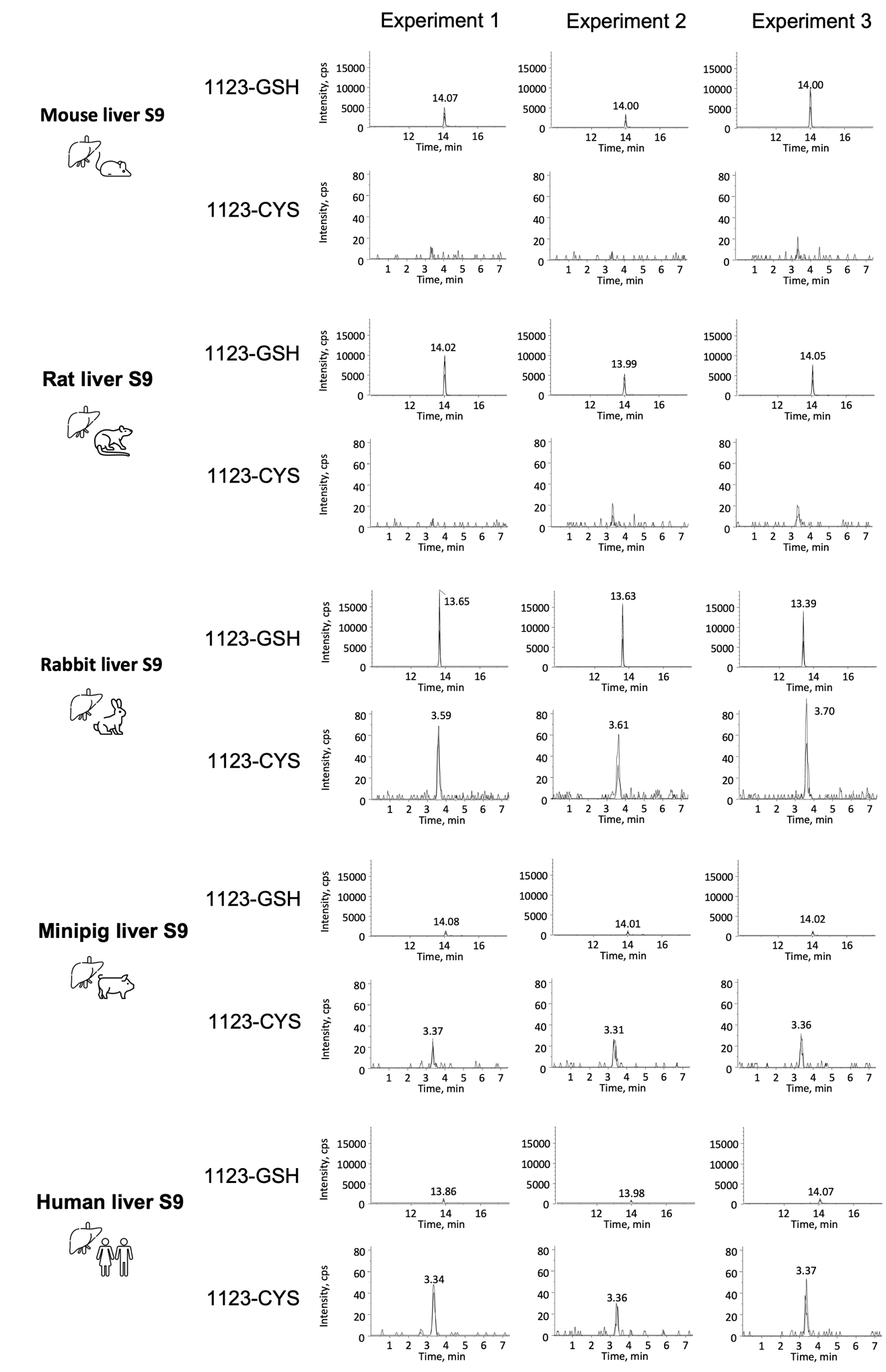


Figure S. 4 LC-MS/MS chromatograms obtained from incubations of HFO-1123 and hepatic S9 fractions of mice, rats, rabbits, minipigs and humans showing signals corresponding to 1123-GSH [m/z Da: 390.1 / 221.0, 390.1 / 118.2, 390.1 / 175.0] and 1123-CYS [m/z Da: 203.8 / 118.1, 203.8 / 164.0].

**Figure S.5**


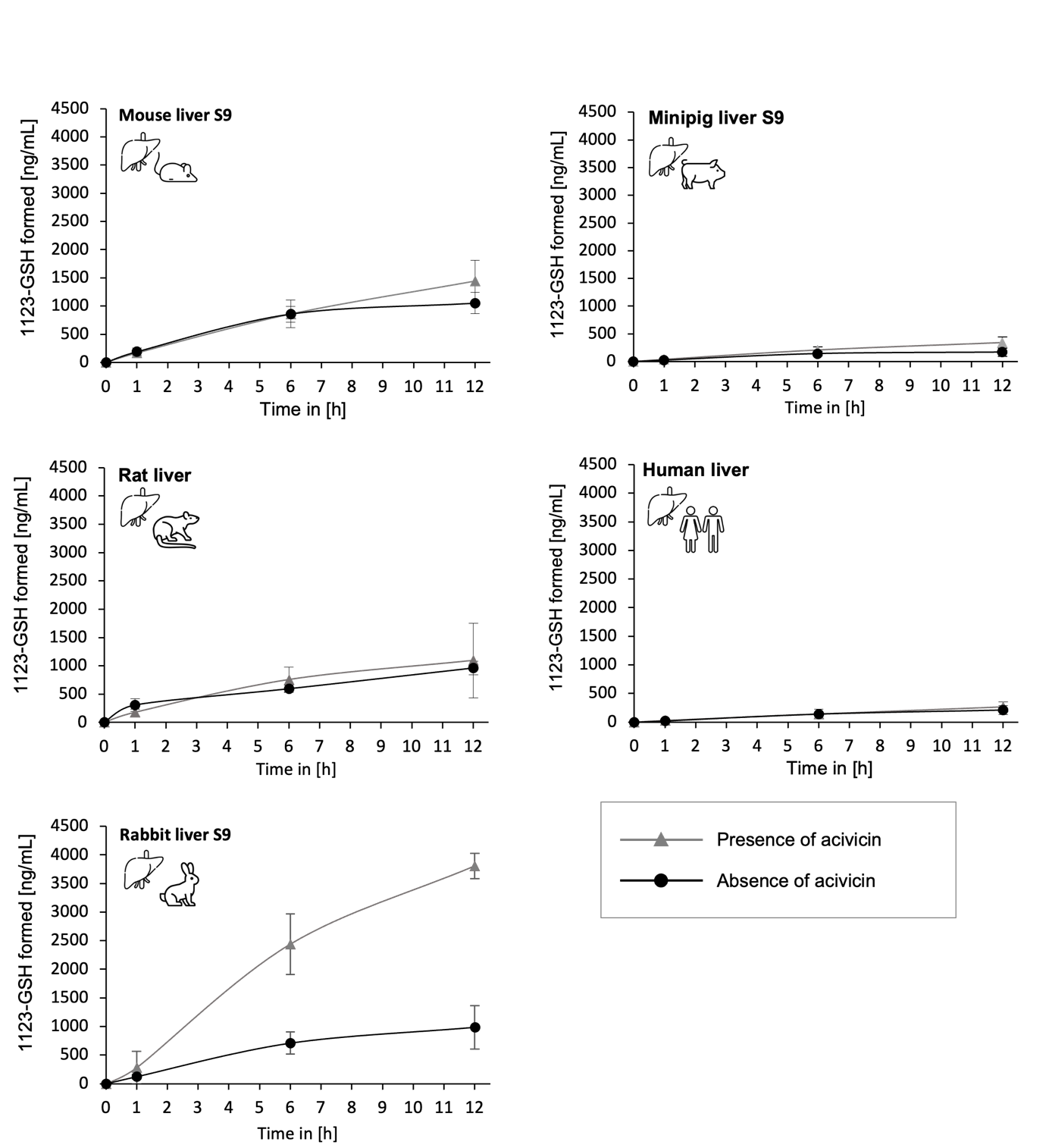


**Figure S. 5** Time-depended formation of 1123-GSH in hepatic S9 fractions of mice, rats, rabbits, minipigs and humans in the presence or absence acivicin. Results are presented as mean ± standard deviation (n=3).

**Figure S.6**


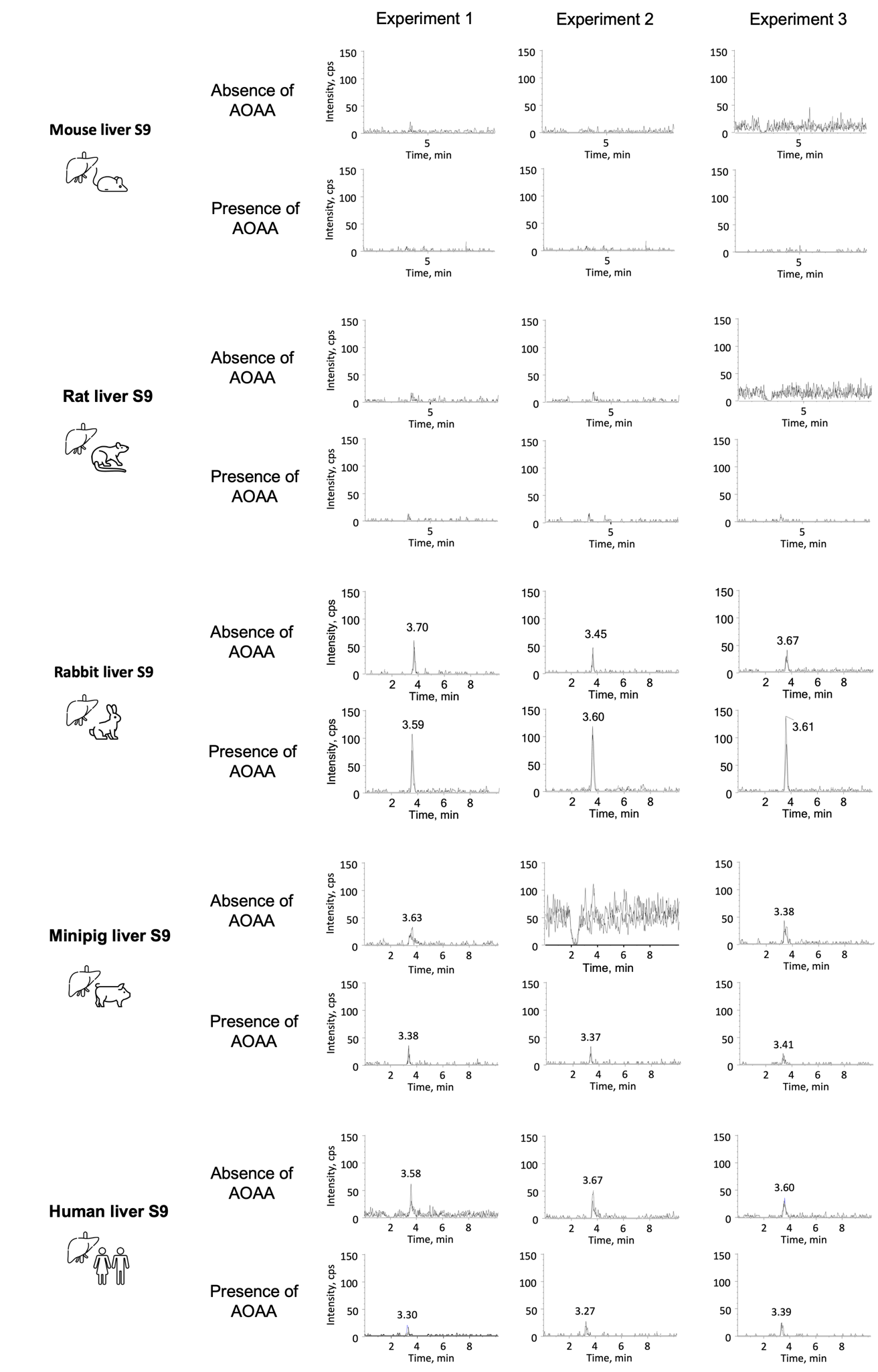


Figure S. 6 4 LC-MS/MS chromatograms obtained from incubations of HFO-1123 and hepatic S9 fractions of mice, rats, rabbits, minipigs and humans showing signals corresponding to 1123-CYS [m/z Da: 203.8 / 118.1, 203.8 / 164.0] in the presence or absence of the β-lyase inhibitor AOAA.
